# Deceleration of single-stranded DNA passing through a nanopore using a nanometre-sized bead structure

**DOI:** 10.1038/srep16640

**Published:** 2015-11-12

**Authors:** Yusuke Goto, Takanobu Haga, Itaru Yanagi, Takahide Yokoi, Ken-ichi Takeda

**Affiliations:** 1 Hitachi Ltd., Central Research Laboratory, 1-280 Higashi-koigakubo, Kokubunji, Tokyo, 185-8603.

## Abstract

DNA sequencing with a solid-state nanopore requires a reduction of the translocation speeds of single-stranded DNA (ssDNA) over 10 μs/base. In this study, we report that a nanometre-sized bead structure constructed around a nanopore can reduce the moving speed of ssDNA to 270 μs/base by adjusting the diameter of the bead and its surface chemical group. This decelerating effect originates from the strong interaction between ssDNA and the chemical group on the surface of the bead. This nanostructure was simply prepared by dip coating in which a substrate with a nanopore was immersed in a silica bead solution and then dried in an oven. As compared with conventional approaches, our novel method is less laborious, simpler to perform and more effective in reducing ssDNA translocation speed.

Nanopore DNA sequencing is undergoing rapid development for its potential advantages (i.e., long reads, low cost and high speed^1^,^2^) compared with existing DNA sequencing methods^3^. Regarding the theoretical method of nanopore DNA sequencing, an external voltage is applied across a nanopore and the ion current flows through the nanopore. When a DNA strand electrophoretically enters the nanopore due to its negative charge, the ion current is partially blocked. Because the blockade current differs for the four nucleotides (A, T, G, and C), the sequence of nucleotides in the DNA strand is determined by measuring the series of changes in the current as the strand translocates through the nanopore. Based on its composition, a nanopore is categorized as either a biological nanopore or a solid-state nanopore. The biological nanopores have already succeeded in the proof-of-principle DNA sequencing and achieved a read length of 4,500 bases^4^,^5^,^6^,^7^. In addition, epigenetic modification (5-methylcytosine and 5-hydroxymethylcytosine) could be detected with Mycobacterium smegmatis porin A (MspA)^8^. Although such significant advancements towards practical DNA sequencing have been achieved for biological nanopores^4^,^5^,^6^,^7^,^8^, solid-state nanopores^1^,^2^,^9^,^10^ are promising because of their higher robustness and the stability of the materials.

One of the primary issues in actualizing solid-state nanopore sequencing, however, is that the DNA translocation through the nanopore is too rapid relative to the sampling rate of the amplifier^11^. In a previous study^12^, among many methods aimed at decelerating DNA^11^,^13^,^14^,^15^,^16^,^17^,^18^ as it passes through a nanopore, we selected a method that narrows the nanopore because it is compatible with the above-mentioned sequencing measurement. This method effectively decelerates single-stranded DNA (ssDNA) translocation up to 1 μs/base^9^,^12^. However, the translocation speed remained too rapid for the sequencing measurement, even though a newly developed rapid amplifier (1 MHz sampling rate)^19^ is used. To obtain more than 10 data points per base, the speed should be slower than 10 μs/base. Accordingly, an additional approach that is compatible with the narrow nanopore method and the sequence measurement is required.

Squires *et al.* recently developed a method to meet these requirements by electrospinning a nanofibre mesh on a substrate with a nanopore^20^. In this method, double-stranded DNA can interact with nanofibres as it translocates through the mesh, and it can be decelerated to approximately 1 μs/base. Thus, to achieve the goal (10 μs/base), optimization of the mesh structure and the chemical properties of the fibres to further reduce the translocation speed is still required. However, the chemical synthesis of the fibre is laborious and time consuming, and electrospinning requires complex and expensive equipment.

To address these issues, we coated nanometre-sized beads on the membrane with a nanopore, as illustrated in Fig. 1. In this approach, densely packed beads provide the nanoporous structure, and the chemical functional group on the surface of the bead interacts with the DNA molecule. Thus, it is expected that our approach will have an effect similar to that of the nanofibre-mesh method. The porous size can be optimized by changing the diameter of the bead; the interaction between ssDNA and the surface of the bead can be enhanced by modifying the chemical group on the bead. Because beads with various diameters and chemical modifications on the surfaces are commercially available, the optimized deceleration effect is easily accomplished. Moreover, the method for coating beads in densely packed structures is straightforward; it consists of dip coating the substrate with the nanopore in a bead suspension solution and drying the membrane^21^,^22^. Our approach is therefore advantageous over the nanofibre-mesh method^20^ in optimizing the deceleration effect.

## Results and Discussion

**Figure 2:**
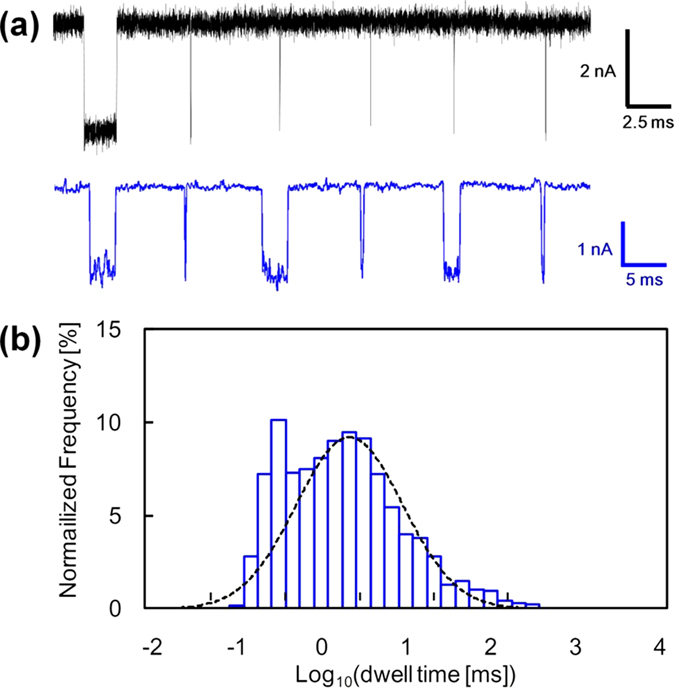
ssDNA translocation through a nanopore on the substrate coated with 100-nm silica beads. (**a**) Typical concatenated translocation events for 5.3 k-mer poly(dA) passing through a nanopore on a bare substrate (upper) and on a substrate coated with 100-nm silica beads (lower). The bare substrate had a 2.0-nm-diameter nanopore in a 20-nm-thickness membrane. The bead-coated substrate had a 3.0-nm-diameter nanopore in 12-nm-thickness membrane. The applied voltage was 1 V. The data was low-pass filtered at 100 kHz (bare substrate) or 5 kHz (bead-coated substrate). (**b**) Log-scaled histogram of dwell time for the same silica-bead-coated substrate (blue, N = 1167). The broken line is a fitted curve using a log-normal distribution.

In this study, we have translocated 5.3 k-mer single-stranded poly(dA)^12^ through nanopores with diameters of 2.0 nm to 3.0 nm in 12-nm- or 20-nm-thickness Si_3_N_4_ membrane. ssDNA translocation events in 1 M KCl solution were measured at an applied bias of high voltage (i.e., 1 V) with data filtered to a 5-kHz bandwidth. At low bias voltage (300 mV), we observed only clogging of a nanopore by ssDNA in the case of bead-coated substrates (see Supplementary Section SI-3). Taking into account that DNA molecules pass through typical silicon nitride pores with the applied voltage of 20 mV^23^, this voltage-dependent phenomenon is probably due to the high energy barrier of the bead structure for ssDNA translocation. Figure 2a shows the blockade events of 5.3 k-mer ssDNA for a bare substrate (upper) and a substrate coated with 100-nm silica beads (lower). The clearly prolonged blockade events for the bead-coated substrate indicate that the translocations of ssDNA through the nanopore were decelerated. The observed blockade currents for the bead-coated substrate (1.9 ± 0.4 nA) were in good agreement with the theoretically calculated value (i.e., 2.2 nA = baseline (10 nA) × (ssDNA diameter (1.4 nm))^2^/(nanopore diameter (3.0 nm))^2^). Moreover, coating beads on nanopore has no impact on noise and I–V characteristics (see Supplementary Section SI-4). Thus, the beads have a negligible effect on the measurement of the blockade current. The log-scaled histogram of the dwell times for the bead-coated substrate is shown in Fig. 2b. The distribution broadened for the longer timescales as compared with that of the bare substrate (see Supplementary Section SI-5). This finding statistically proves that the ssDNA translocation was decelerated by the beads. The histogram has indeed two populations: the longer one was well fitted to a single log-normal distribution^24^; the shorter one was the tale part of the same log-normal distribution as that observed in the bare substrate (Figure S11). The main part of this distribution was cut off by the low-pass filter and only the tale part left; the short events attenuated by the filter were included in this tale part (Supplementary Section SI-5). Such a bimodal distribution was also observed for the nanofibre-mesh-coated substrates developed by Squires *et al.*^20^. Thus, it can be similarly predicted that shorter and longer dwell-time populations correspond to ssDNA translocations that are unaffected and affected by the beads, respectively. We designate the shorter and longer dwell-time populations as “short” and “long”. We use “long” events to measure the deceleration effect of beads on ssDNA translocation.

**Figure 3:**
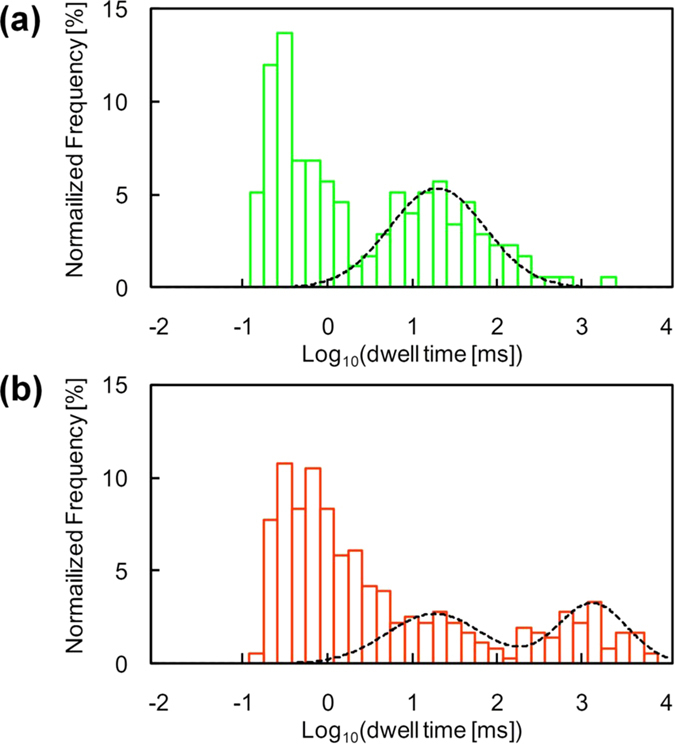
Histograms of translocation time for 5.3 k-mer poly(dA) passing through a nanopore. (**a**) 50-nm silica bead-coated substrate (N = 175) and (**b**) 50-nm amine-silica bead-coated substrate (N = 362). Nanopore diameter and membrane thickness in each substrate were 2.0 nm and 20 nm, respectively. Translocation events occurred when the applied voltage was 1 V (low-pass filtered at 5 kHz). Distributions of both substrates were also fitted successfully using log-normal distributions.

To further characterize the “long” event populations, we measured the ssDNA translocation using beads of various sizes and containing various functional groups. Figure 3 shows the log-scaled histogram of dwell times measured using substrates coated with 50-nm plain (i.e., silanol-functionalized) and amine-functionalized silica beads. In both cases, the histograms also have “short” and “long” event populations. The “long” populations were well fitted to a single log-normal distribution for the silica beads and two log-normal distributions for the amine-silica beads. The “long” distribution in both cases of the 50-nm beads was slower than that for the 100-nm beads. This finding indicates that the smaller nanoporous structure increases the deceleration effect of ssDNA translocation because of the increased probability of ssDNA collision with the beads^21^.

Moreover, the finding that the “long” event population for the amine-silica beads broadens for longer timescales compared with that for silica beads (Fig. 3b) agrees with the results of previous AFM studies^25^,^26^. The studies demonstrated that DNA interacts more strongly with an amine group^26^ than with a silanol group^25^. Therefore, the longer population in the two “long” events for the amine-silica bead-coated substrate is attributable to the stronger interaction between ssDNA and an amine group. The shorter population in the two “long” event populations probably stems from ssDNA translocations decelerated by the silanol group remaining on the surface of the bead because the characteristic dwell time (18 ms) is consistent with that of plain silica beads (17 ms). Moreover, amine-silica beads are generally prepared by chemically modifying plain silica beads with amine groups, and the modification may be incomplete. The remaining silanol group should be excluded in the improved modification process (for example, using aminoalkyl-alkoxysilane) to reduce the variation in translocation speeds. Therefore, it is concluded that the interaction between ssDNA and the chemical group on the bead surface creates the dragging force that decelerates the translocation of ssDNA through a nanopore.

**Figure 4:**
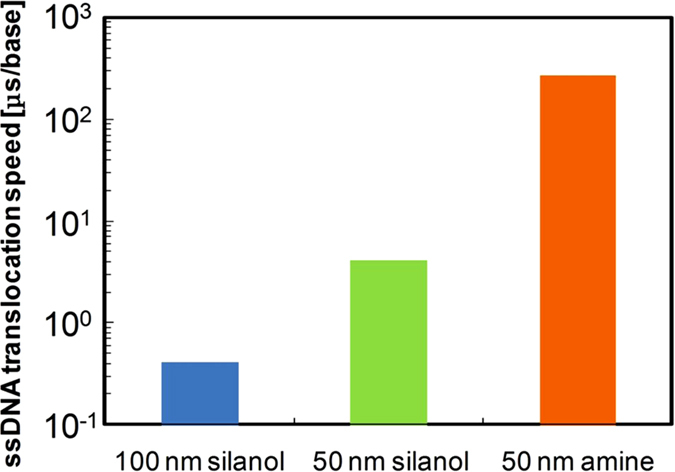
Comparison chart of peak dwell times of the slowest distribution observed for three different bead-coated substrates: 100-nm silanol-silica bead (blue), 50-nm silanol-silica bead (green) and 50-nm amine-silica bead (orange).

Figure 4 shows the translocation times of ssDNA for the following three bead-coated substrates: a 100-nm silanol-silica bead, 50-nm silanol-silica bead, and 50-nm amine-silica bead. The values were obtained by converting the characteristic dwell times of “long” event populations so that their units were microseconds per base. The reduction of the diameter of the bead (from 100 nm to 50 nm) and the change of the surface chemical group (from silanol to amine) resulted in the slowest translocation speed of 270 μs/base, which is well above the target translocation speed (10 μs/base). Although direct comparison with the results of the nanofibre mesh (1 μs/base^20^) is difficult because double-stranded DNA was used, we strongly believe that our system can provide a larger deceleration effect.

**Figure 5:**
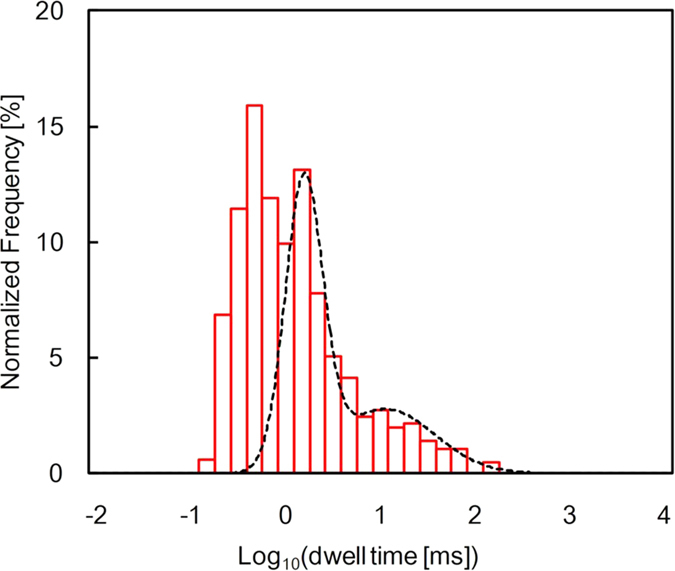
Histogram of dwell time for short 60-mer poly(dA) translocation using 50-nm amine-silica bead-coated substrates. Translocation events (N = 656) were obtained under the condition that the applied voltage was 1 V (low-pass filtered at 5 kHz). Nanopore diameter and membrane thickness were 2.0 nm and 20 nm, respectively. The distribution was fitted successfully using log-normal distributions.

Given that the interaction with the surface of the bead determined the ssDNA translocation speed, it is expected that the deceleration effect of the bead structure is dependent on the base length. Therefore, we conducted the translocation experiment on short ssDNA (60-mer poly(dA)) using the same 50-nm amine-silica bead-coated substrate. Figure 5 presents the histogram of the 60-mer poly(dA) translocation time per base, which was similarly fitted to two log-normal distributions. The ssDNA translocation speed derived from “long” event populations was 160 μs/base, which was slightly faster than that of the 5.3 k-mer poly(dA) (270 μs/base). The reason for the base-length dependency could be explained as follows. Assuming that the length per base of ssDNA is approximately 0.6 nm/base^27^, the length of the 60-mer poly(dA) is calculated as around 36 nm which is enough to interact with beads (In a close-packed structure of 50-nm beads, the maximum interspace size of the 50-nm bead structure is approximately 17 nm (= (√3 − 1) × 25 nm)). However, compared with 5.3 k-mer poly(dA), the shorter length of 60-mer poly(dA) decreases in the contact probability and led to a faster translocation speed. Nevertheless, the translocation speed of the 60-mer ssDNA sufficiently exceeds the target speed (10 μs/base). This result indicates that our approach can function well as a deceleration method for various lengths of ssDNA.

In conclusion, by adequately selecting the diameter of the bead and its surface chemical group, our nanosized-bead structure can provide a superior deceleration effect compared with the conventional method^20^. Moreover, the achieved DNA translocation speed exceeds that required for practical DNA sequencing (10 μs/base). DNA translocation that was not decelerated, however, remained. Such an unslowed translocation was attributable to unadsorption of DNA on the surface of the bead due to the existence of an imperfectly packed bead structure. To maximize the effect of the bead structure, it will be necessary to reduce the proportion of DNA that was not decelerated from passing by, thus improving the bead packing and chemical treatment. Additionally, it is preferable to uniform the translocation speed in the bead coating system for practical DNA sequencing. In the future, we will try to reduce the broad dwell times by filling a gap between the beads with the cationic polymer (such as poly-L-lysine) using polymer-grafted silica beads^28^. We strongly believe that our deceleration technology will enable direct DNA sequencing in combination with a high-speed amplifier.

## Methods

### Nanopore Fabrication

Substrates with a 12-nm- or 20-nm-thick Si_3_N_4_ membrane (with a small square area within (500 × 500 nm^2^)) were prepared, as previously reported (see Supplementary Section SI-1)^29^. The motion of the coated beads was confined to this square hole, which stabilized the bead structures. Before coating, a nanopore with a diameter of 2.0 to 3.0 nm on the membrane (shown in Supplementary Section SI-1) was fabricated using a transmission electron microscope (HD2700B, Hitachi High-Technologies, Japan) or our previously reported method, dielectric breakdown via multilevel pulse-voltage injection (MPVI)^29^. MPVI was performed as follows. Two chambers (cis and trans chambers) were formed in a custom-made flow cell. Both chambers were filled with 1 M KCl aqueous solution. Two Ag/AgCl electrodes (cis and trans electrodes) were immersed in the solutions and were connected to a pulse-voltage generator (41501B SMU and pulse generator expander, Agilent Technologies, Santa Clara, CA) and an ammeter (4156B Precision semiconductor analyser, Agilent Technologies, Santa Clara, CA). The MPVI procedure was controlled by a program written using Excel Visual Basic for Applications (VBA). The applied voltage and pulse duration were optimized so that a nanopore with the desired diameter was fabricated. After completion of MPVI, the substrates were washed with DI water and dried at room temperature.

### Bead coating

The bead coating was placed on the substrate with a nanopore by dip coating as follows. Before the dip coating, the substrates were cleaned and hydrophilized on each side with argon/oxygen plasma (PA-1, SAMCO, Japan) at 10 W, a flow rate of 20 sccm, and a pressure of 20 Pa for 45 s. The substrates were dipped into an aqueous silica-bead solution (Micromod, Germany) and pulled out of the solution at a speed of approximately 1 mm/s. Various types of silica beads were used, as follows: a 100-nm plain (silanol-functionalized) silica bead, 50-nm plain silica bead, and 50-nm amine-functionalized silica bead. Each bead solution with the desired concentration was prepared by dilution with DI water. After being removed from the solution, the substrates were dried in an oven (DO-300PA, AS ONE, Japan) at 120 °C for 10 minutes. In this manner, a bead-coated substrate was prepared. In the dipping process, the concentration of the silica-bead solution was optimized such that the membranes were fully covered with densely packed beads. This covered condition was confirmed by scanning electron microscopy (SEM, S-5200, Hitachi High-Technologies, Japan) at 5 kV. Figures S3–S5 (shown in Supplementary Section SI-2) are the SEM images of silica-bead-coated substrates with various bead concentrations. The membrane area is indicated by the dashed line. The bead coverage of the membrane increased with increasing bead concentration in all the cases. Under the fully covered condition, the beads were closely packed on the membrane. This packing stabilizes the nanostructure to prevent the beads from re-dispersing into the solution; moreover, the closely packed state provides smaller and more uniform spaces between the beads compared with the sparsely coated state. This bead configuration therefore provides suitable nanoporous spaces to decelerate the ssDNA.

### ssDNA blockade current measurements

The experimental setup was as follows. The prepared substrates were assembled in the same flow cell as that used for the MPVI method. Two chambers (a cis chamber and a trans chamber, each with a volume of 90 μL), which were separated by the substrate, were formed in the flow cell. Both chambers were filled with an aqueous solution consisting of 1 M potassium chloride. Then, 5.3 ± 0.4 k-mer poly(dA) was synthesized as previously reported^12^, and 60-mer poly(dA) was purchased from Nihon Gene Research Laboratory. Only the solution in the cis chamber contained single-stranded poly(dA) with a concentration of 1.7 nM (5.3 k-mer) and 70 nM (60-mer). An Ag/AgCl electrode was immersed in each solution to ensure electrical contact between the chambers.

To detect ssDNA translocation events, a patch-clamp amplifier (Axopatch 200B, Axon Instruments, Union City, CA) was used to apply a voltage of 1 V and to detect the ionic current through the nanopore. The detected current was first low-pass filtered through a four-pole Bessel filter with a cutoff frequency of 5 kHz, then it was digitized with an NI 9223 DAQ AD converter (National Instruments, Austin, TX) at 50 kHz and, subsequently, recorded onto the hard disk of a personal computer. Current blockade events were identified and analysed using OpenNanopore, an open source software package^30^. The entire procedure was performed at room temperature. Figure S6 (see Supplementary Section SI-3) shows a typical time trace of the ion current for a 100-nm silica-bead-coated substrate (nanopore diameter: 3.0 nm, membrane thickness: 20 nm). The blockade events in Figure S6 represent 5.3 k-mer ssDNA passage.

## Additional Information

**How to cite this article**: Goto, Y. *et al.* Deceleration of single-stranded DNA passing through a nanopore using a nanometre-sized bead structure. *Sci. Rep.* **5**, 16640; doi: 10.1038/srep16640 (2015).

## Supplementary Material

Supplementary Information

## Figures and Tables

**Figure 1:**
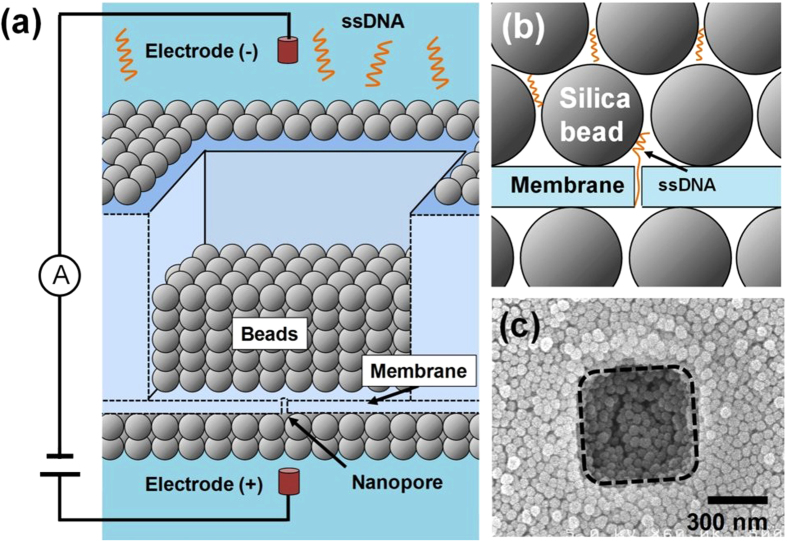
Nanopore with nanometre-sized bead nanostructure. (**a**) Schematic of solid-state nanopore with nanometre-sized bead structure coated on the membrane. (**b**) Schematic showing interaction between ssDNA and the silica bead around a nanopore. (**c**) Typical top-view scanning electron microscope image of the bead-coated substrate. The dashed line shows the square area that is the thinnest part of the membrane. (The thickness of the membrane: 20 nm, the diameter of the beads: 50 nm, the width of the square area: 500 nm, the base length of ssDNA: 60-mer.).

## References

[b1] Dekker C. Solid-state nanopores. Nature Nanotechnol. 2, 209–215 (2007). 18654264 10.1038/nnano.2007.27

[b2] Branton D. *et al.* The potential and challenges of nanopore sequencing. Nature Biotechnol. 26, 1146–1153 (2008). 18846088 10.1038/nbt.1495 PMC2683588

[b3] Metzker M. L. Sequencing technologies - the next generation. Nature Rev. Genet. 11, 31–46 (2010). 19997069 10.1038/nrg2626

[b4] Manrao E. A. *et al.* Reading DNA at single-nucleotide resolution with a mutant MspA nanopore and phi29 DNA polymerase. Nature Biotechnol. 30, 349–353 (2012). 22446694 10.1038/nbt.2171 PMC3757088

[b5] Ayub M. & Bayley H. Individual RNA base recognition in immobilized oligonucleotides using a protein nanopore. Nano lett. 12, 5637–5643 (2012). 23043363 10.1021/nl3027873 PMC3505278

[b6] Cherf G. M. *et al.* Automated forward and reverse ratcheting of DNA in a nanopore at 5-Å precision. Nature Biotechnol. 30, 344–348 (2012). 22334048 10.1038/nbt.2147 PMC3408072

[b7] Laszlo A. H. *et al.* Decoding long nanopore sequencing reads of natural DNA. Nature Biotechnol. 32, 829–833 (2014). 24964173 10.1038/nbt.2950 PMC4126851

[b8] Laszlo A. H. *et al.* Detection and mapping of 5-methylcytosine and 5-hydroxymethylcytosine with nanopore MspA. Proc. Natl. Acad. Sci. USA 110, 18904–18909 (2013). 24167255 10.1073/pnas.1310240110 PMC3839702

[b9] Venta K. *et al.* Differentiation of short, single-stranded DNA homopolymers in solid-state nanopores. ACS Nano 7, 4629–4636 (2013). 23621759 10.1021/nn4014388 PMC3724363

[b10] Xie P., Xiong Q., Fang Y., Qing Q. & Lieber C. M. Local electrical potential detection of DNA by nanowire–nanopore sensors. Nature Nanotechnol. 7, 119–125 (2012). 22157724 10.1038/nnano.2011.217 PMC3273648

[b11] Venkatesan B. M. & Bashir R. Nanopore sensors for nucleic acid analysis. Nature Nanotech. 6, 615–624 (2011). 10.1038/nnano.2011.129 21926981

[b12] Akahori R. *et al.* Slowing single-stranded DNA translocation through a solid-state nanopore by decreasing the nanopore diameter. Nanotechnology 25, 275501 (2014). 24960034 10.1088/0957-4484/25/27/275501

[b13] Fologea D., Uplinger J., Thomas B., McNabb D. S. & Li J. Slowing DNA translocation in a solid-state nanopore. Nano Lett. 5, 1734–1737 (2005). 16159215 10.1021/nl051063o PMC3037730

[b14] Mirsaidov U., Comer J., Dimitrov V., Aksimentiev A. & Timp G. Slowing the translocation of double-stranded DNA using a nanopore smaller than the double helix. Nanotechnology 21, 395501 (2010). 20808032 10.1088/0957-4484/21/39/395501 PMC3170403

[b15] Kowalczyk S. W., Tuijtel M. W., Donkers S. P. & Dekker C. Unraveling single-stranded DNA in a solid-state nanopore. Nano lett. 10, 1414–1420 (2010). 20235508 10.1021/nl100271c

[b16] Wanunu M., Sutin J., McNally B., Chow A. & Meller A. DNA translocation governed by interactions with solid-state nanopores. Biophys. J. 95, 4716–4725 (2008). 18708467 10.1529/biophysj.108.140475 PMC2576395

[b17] Singer A., Kuhn H., Frank-Kamenetskii M. & Meller A. Detection of urea-induced internal denaturation of dsDNA using solid-state nanopores. J. Phys. Condens. Matter 22, 454111 (2010). 21339599 10.1088/0953-8984/22/45/454111

[b18] Wanunu M., Morrison W., Rabin Y., Grosberg A. Y. & Meller A. Electrostatic focusing of unlabelled DNA into nanoscale pores using a salt gradient. Nature Nanotechnol. 5, 160–165 (2010). 20023645 10.1038/nnano.2009.379 PMC2849735

[b19] Rosenstein J. K., Wanunu M., Merchant C. A., Drndic M. & Shepard K. L. Integrated nanopore sensing platform with sub-microsecond temporal resolution. Nature Methods 9, 487–492 (2012). 22426489 10.1038/nmeth.1932 PMC3648419

[b20] Squires A. H., Hersey J. S., Grinstaff M. W. & Meller A. A nanopore–nanofiber mesh biosensor to control DNA translocation. J. Am. Chem. Soc. 135, 16304–16307 (2013). 24143914 10.1021/ja408685x PMC4039743

[b21] Zeng Y. & Harrison D. J. Self-Assembled Colloidal Arrays as Three-Dimensional Nanofluidic Sieves for Separation of Biomolecules on Microchips. Anal. Chem. 79, 2289–2295 (2007). 17302388 10.1021/ac061931h

[b22] Zhang H. & Wirth M. J. Electromigration of Single Molecules of DNA in a Crystalline Array of 300-nm Silica Colloids. Anal. Chem. 77, 1237–1242 (2005). 15732902 10.1021/ac0488964

[b23] Fologea D. *et al.* Slowing DNA Translocation in a Solid-State Nanopore. Nano Lett. 5, 1734–1737 (2005). 16159215 10.1021/nl051063o PMC3037730

[b24] Larkin J. *et al.* Slow DNA Transport through Nanopores in Hafnium Oxide Membranes. ACS Nano 7, 10121–10128 (2013). 24083444 10.1021/nn404326f PMC4729694

[b25] Kühner F. *et al.* Friction of Single Polymers at Surfaces. Langmuir 22, 11180–11186 (2006). 17154600 10.1021/la061704a

[b26] Erdmann M., David R., Fornof A. & Gaub H. E. Electrically controlled DNA adhesion. Nature Nanotechnol. 5, 154–159 (2010). 20023647 10.1038/nnano.2009.377

[b27] Murphy M. C. *et al.* Probing single-stranded DNA conformational flexibility using fluorescent spectroscopy. Biophys. J. 86, 2530–2537 (2004). 15041689 10.1016/S0006-3495(04)74308-8 PMC1304100

[b28] Kar M., Vijayakumar P. S., Prasad B. L. V. & Gupta S. S. Synthesis and Characterization of Poly-L-lysine-Grafted Silica Nanoparticles Synthesized via NCA Polymerization and Click Chemistry. Langmuir 26, 5772–5781 (2010). 20337478 10.1021/la903595x

[b29] Yanagi I., Akahori R., Hatano T. & Takeda K. Fabricating nanopores with diameters of sub-1 nm to 3 nm using multilevel pulse-voltage injection. Sci. Rep. 4, 5000 (2014). 24847795 10.1038/srep05000 PMC4028839

[b30] Raillon C. *et al.* Fast and automatic processing of multilevel events in nanopore translocation experiments. Nanoscale 4, 4916–4924 (2012). 22786690 10.1039/c2nr30951c

