# Supplementary material for: Deceleration of single-stranded DNA passing through a nanopore using a nanometre-sized bead structure
Source: Sci Rep. 2015 Nov 12;5:16640. doi: 10.1038/srep16640 (PMC4642329; doi:10.1038/srep16640)
Supplement: Supplementary Information [file srep16640-s1.doc]

**Supplementary Information**

**Deceleration of single-stranded DNA passing through**

**a nanopore using a nanometre-sized bead structure**

Yusuke Goto*, Takanobu Haga, Itaru Yanagi, Takahide Yokoi and Ken-ichi Takeda

Hitachi Ltd., Central Research Laboratory,

1-280 Higashi-Koigakubo, Kokubunji, Tokyo, 185-8603

**The supplementary information includes:**

**SI-1. Substrate with a membrane designed for nanopore fabrication**

**SI-2. SEM images of a substrate coated with several silica beads**

**SI-3. Time trace of the ionic current and typical ssDNA blockade events**

**SI-4. Noise and I-V characteristics of nanopores coated with silica beads**

**SI-5. Dwell time of ssDNA translocation for bare substrates**

**SI-6. Data reproducibility**

**SI-1. Substrate with a membrane designed for nanopore fabrication**

Figure S1 shows schematic image of a substrate with a membrane for nanopore fabrication. Typical nanopores drilled by a TEM beam are shown in Figure S2.


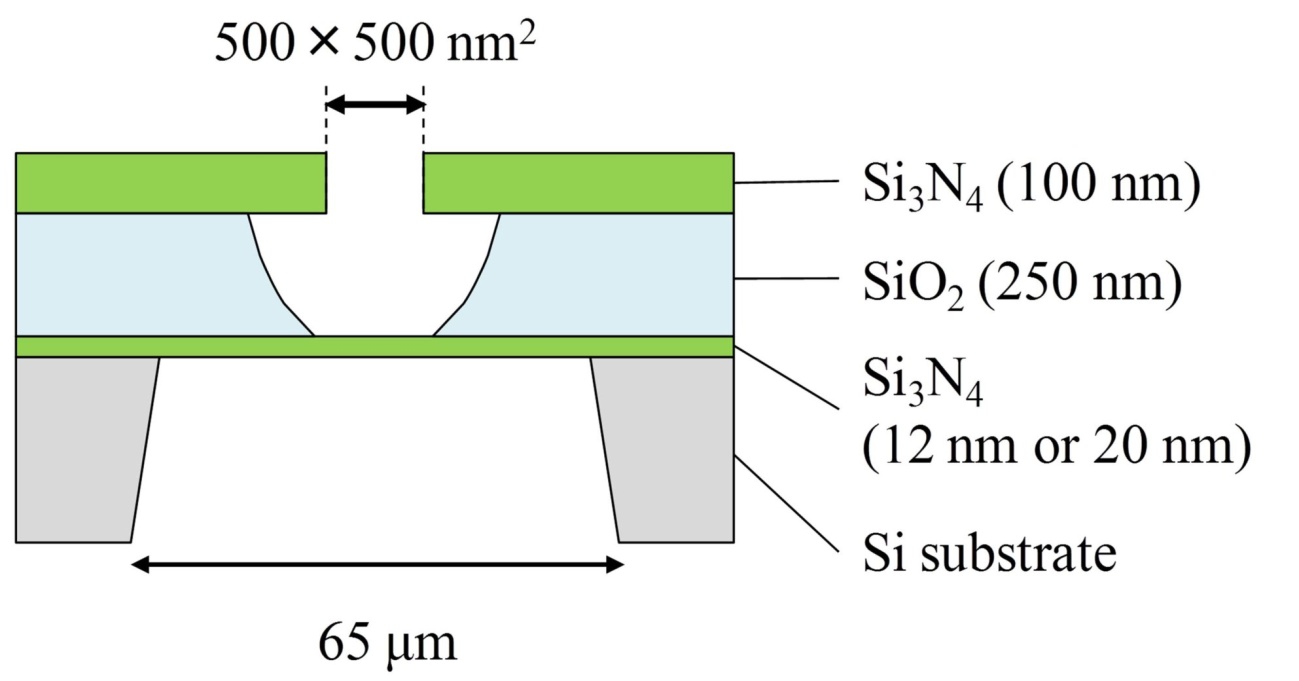


**Figure S1.** Substrate with a membrane designed for nanopore fabrication. 100-nm-thick Si_3_N_4_ layer, 250-nm-thick SiO_2_ layer, and a 12-nm- or 20 nm-thick Si_3_N_4_ layer were deposited on Si substrates. A square hole (500 × 500 nm^2^) was fabricated by dry etching, and the SiO_2_ layer was partially eliminated by HF etching.


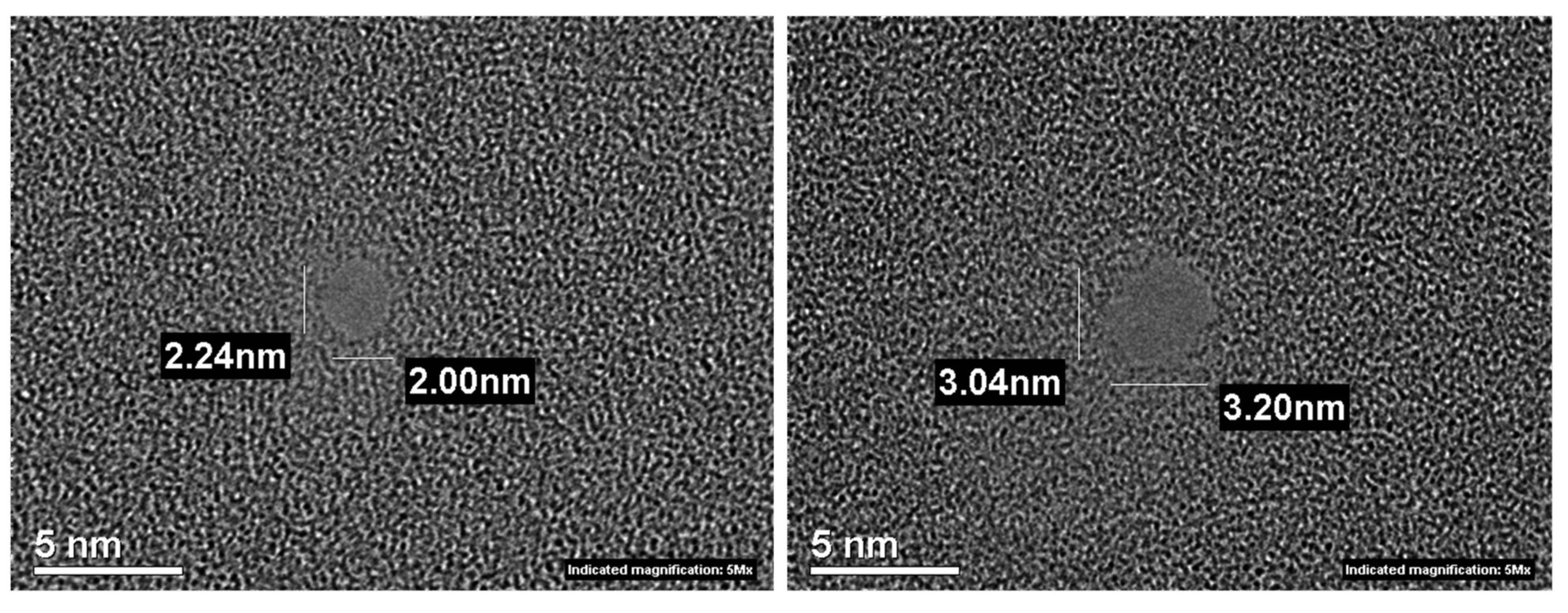


**Figure S2.** Typical TEM images of a nanopore drilled by a focused TEM beam.

**SI-2. SEM images of a substrate coated with silica beads**

Figure S3-S5 show typical SEM images of substrates coated with several silica beads.


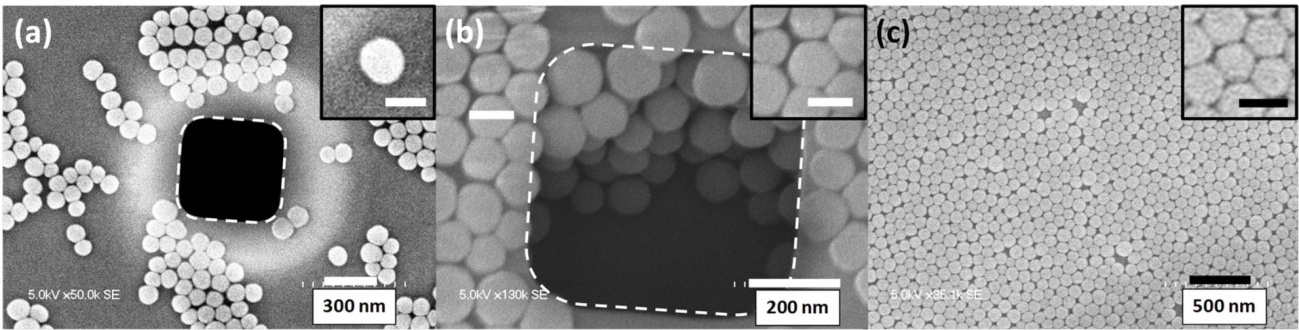


**Figure S3.** Top-view SEM images of a substrate coated with 100-nm silica beads: bead concentrations of (a) 2.4×10^9^/μL, (b) 6.0×10^9^/μL, and (c) 6.8×10^9^/μL. The square area is surrounded by a dashed line. The insets show magnified views of typical beads. Inset scale bar: 100 nm.


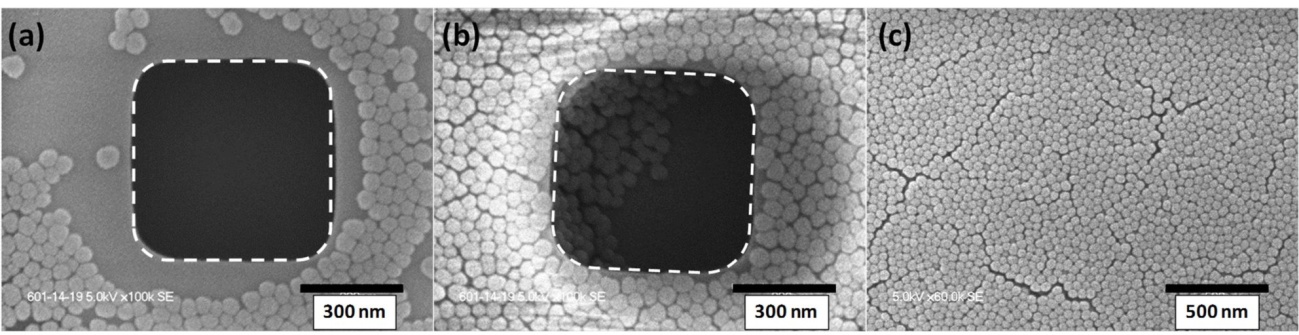


**Figure S4.** Top view SEM images of substrate coated with 50-nm silica beads: bead concentrations of (a) 2.7×10^10^/μL, (b) 6.3×10^10^/μL, and (c) 1.9×10^11^/μL. The square area is surrounded by a dashed line.


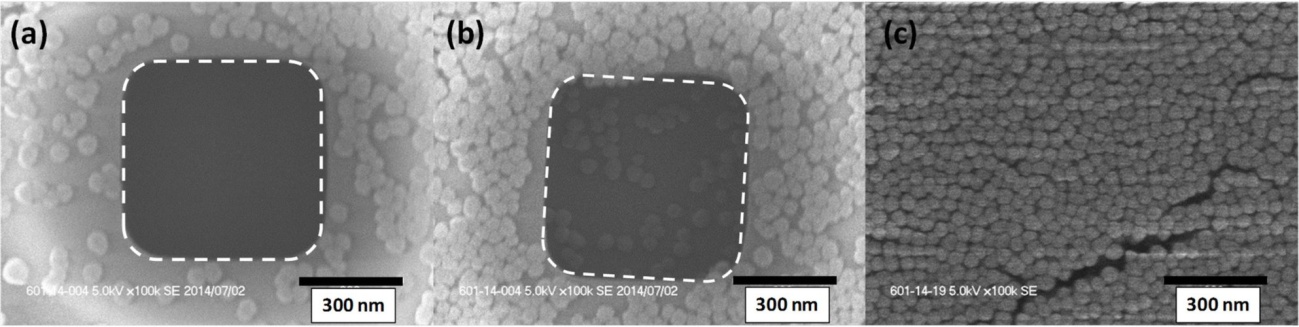


**Figure S5.** Top-view SEM images of substrate coated with 50-nm amine-silica beads: bead concentrations of (a) 1.9×10^10^/μL, (b) 2.7×10^10^/μL, and (c) 3.8×10^11^/μL. The square area is surrounded by a dashed line.

**SI-3. Time trace of the ionic current and typical ssDNA blockade events**


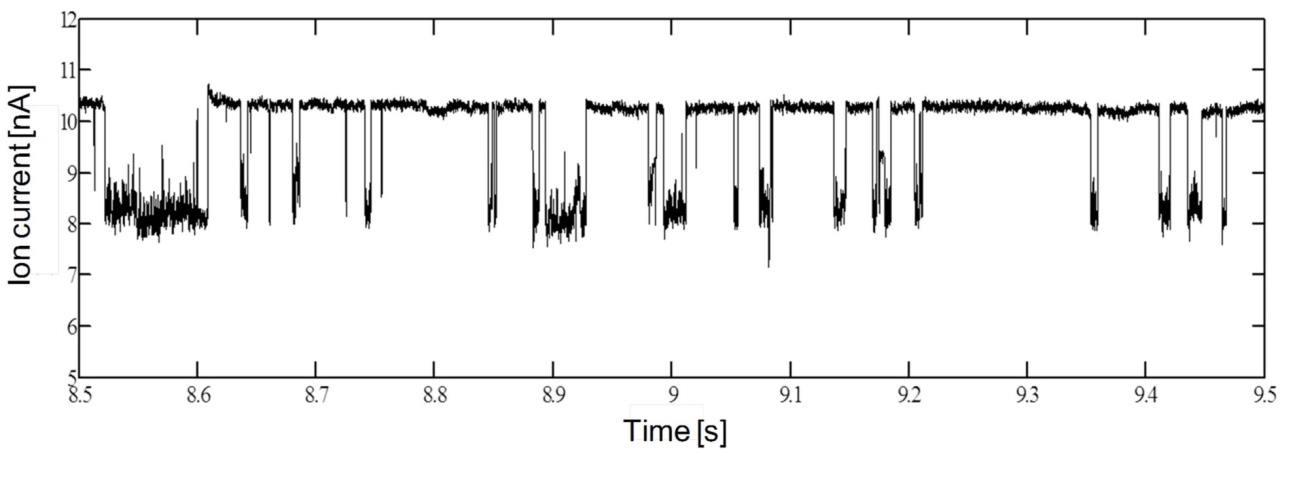


**Figure S6.** Typical time trace of the ionic current for a 100-nm bead-coated substrate (nanopore diameter: 3.0 nm, membrane thickness: 20 nm). Blocakde events of 5.3 k-mer poly(dA) was measured in 1 M KCl aqueous solution. The applied voltage was 1 V (low-pass filtered at 5 kHz).

**
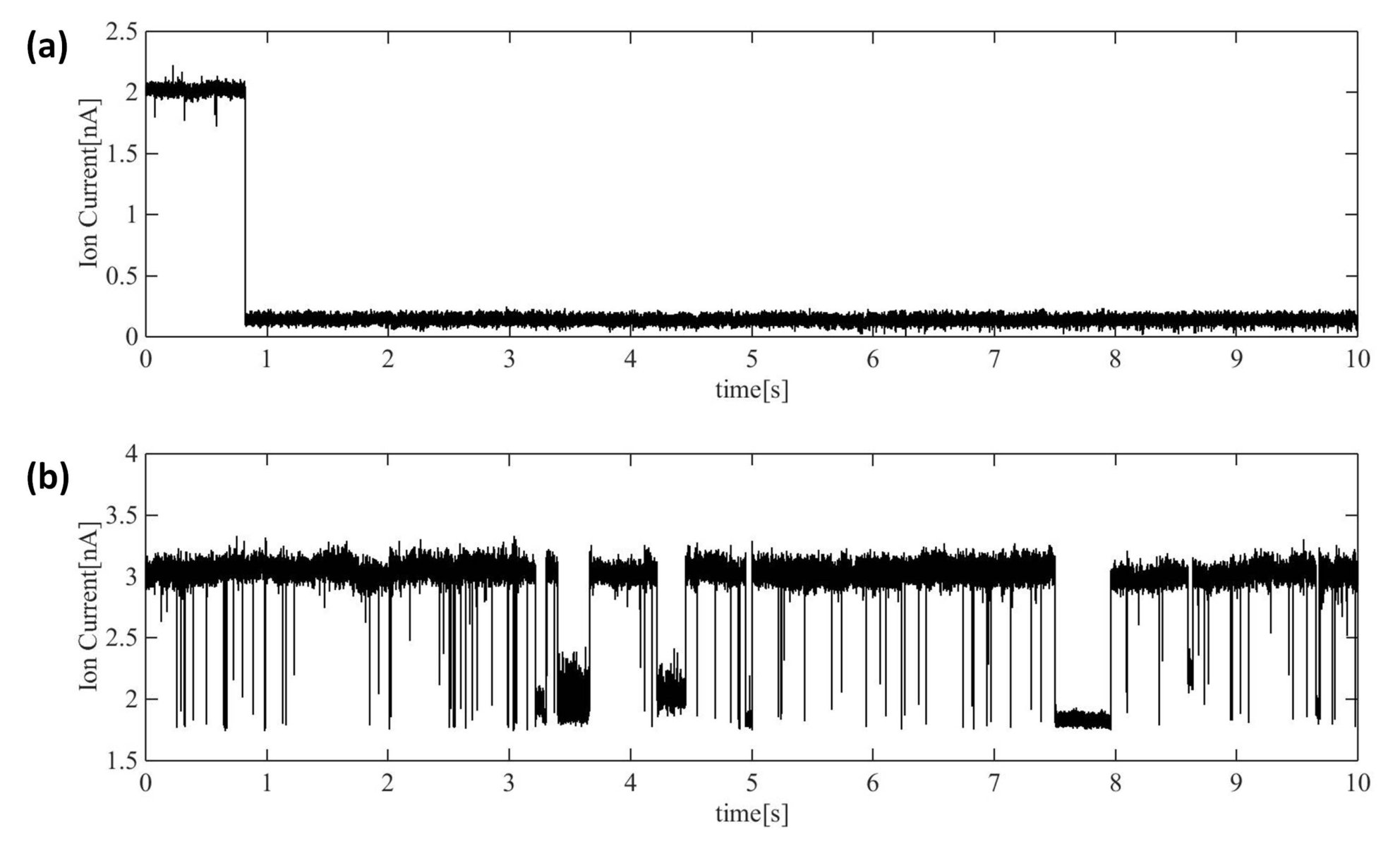
**

**Figure S7.** Typical time traces of the ionic current for 50-nm bead-coated substrates at low voltage (a) (0.3 V, nanopore diameter: 2.0 nm, membrane thickness: 12 nm) and at high voltage (b) (1 V, nanopore diameter: 2.0 nm, membrane thickness: 20 nm). The ionic current rapidly dropped within few seconds at low voltage.

**SI-4. Noise and I-V characteristics of nanopores coated with silica beads**

Noise spectra of nanopores coated with silica beads were shown in Figure S8 (low-pass filtered at 5 kHz) and Figure S9 (low-pass filtered at 1 MHz). I-V characteristic was also shown in Figure S10. The data reveals that the bead coating layer did not substantially affect the noise behaviour and the conductance of a nanopore.


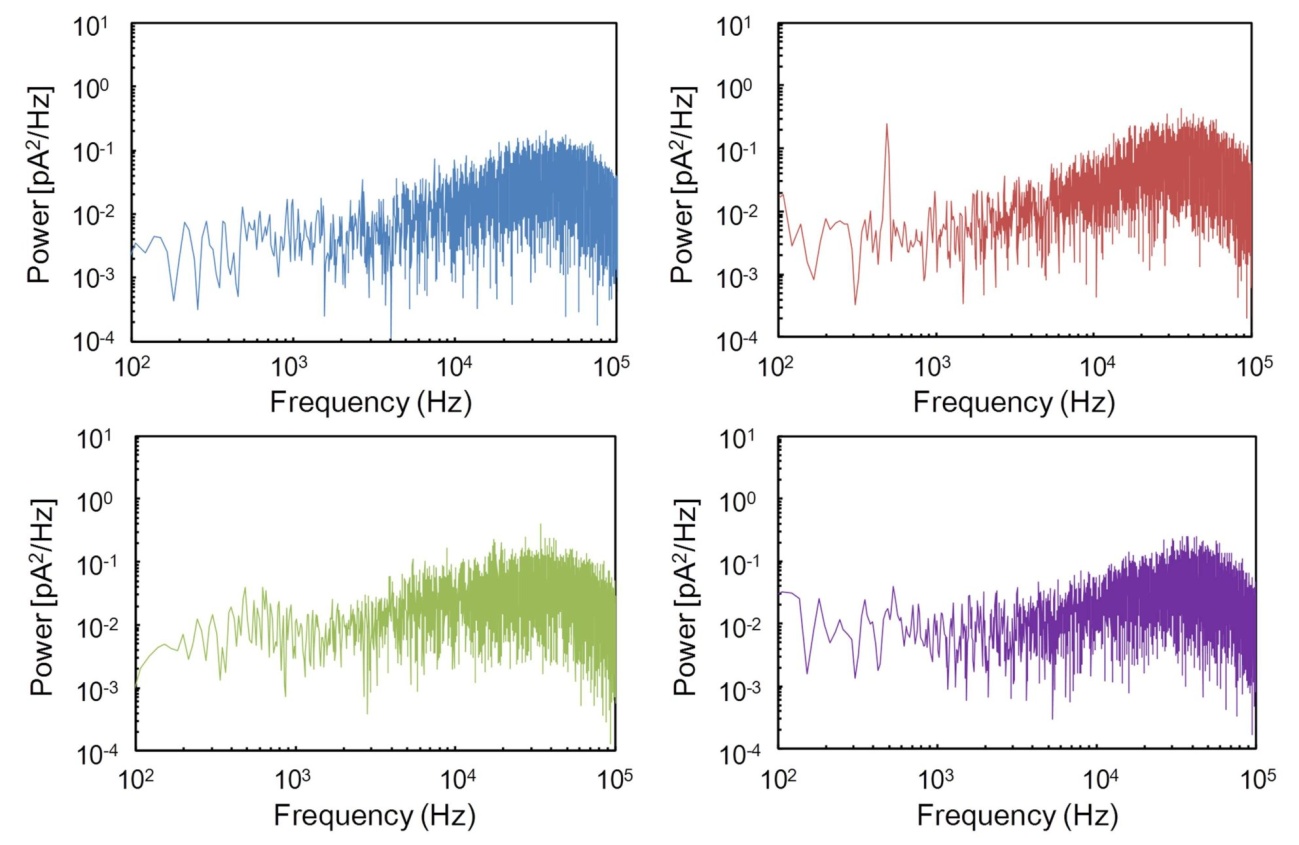


**Figure S8.** Current power spectral density of the same nanopores, shown in Figure S11 and Figure 2-4, on the bare substrate (blue), the 100-nm silica bead-coated substrate (red), the 50-nm silica bead-coated substrate (green) and the 50-nm amine-silica bead-coated substrate (purple). The ionic current noise was measured at 0 V in 1 M KCl aqueous solution using a patch-clamp amplifier Axopatch 200B. The detected currents were low-pass filtered with a cut-off frequency of 5 kHz.

**
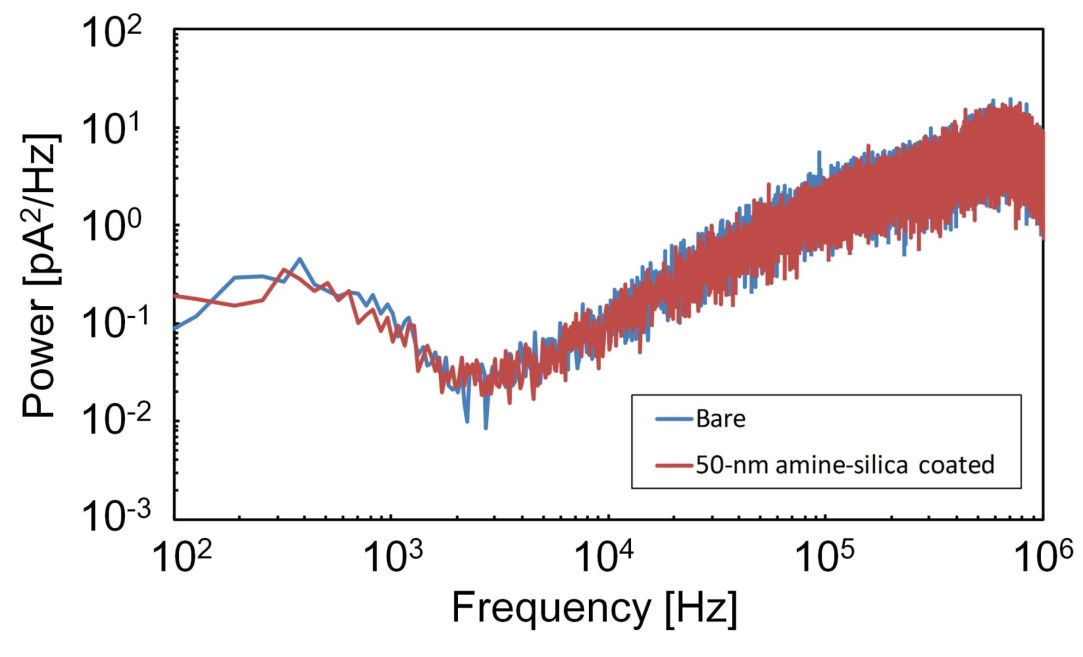
**

**Figure S9.** Current power spectral density of nanopores on the bare substrate (blue) and the 50-nm amine-silica bead-coated substrate (red) in the high frequency regime. The bare substrate had a 1.6-nm-diameter nanopore in a 12-nm-thickness membrane. The bead-coated substrate had a 2.0-nm-diameter nanopore in a 12-nm-thickness membrane. The ionic current noise was measured at 0 V in 1 M KCl aqueous solution using a 1-MHz high-speed amplifier VC100 (Chimera Instruments, New York, NY, USA). The detected currents were low-pass filtered with a cut-off frequency of 1 MHz.

**
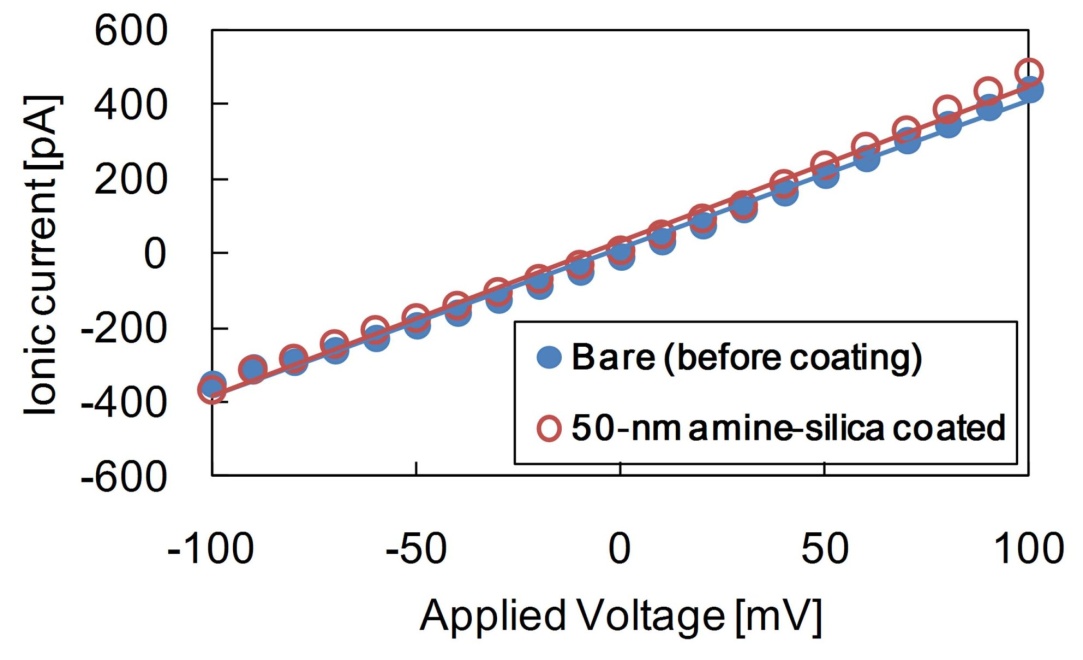
**

**Figure S10.** Current-voltage characteristic of the same nanopore before bead-coating (blue) and after the 50-nm amine-silica bead-coating (red). The substrate had a 1.4-nm-diameter nanopore in a 12-nm-thickness membrane. The ionic current was measured in 1 M KCl aqueous solution.

**SI-5. Dwell time of ssDNA translocation for bare substrates**

In the main paper, we performed all the experiments using 5-kHz filter with low sampling rate (50 kHz) because the event detection time of long duration events with high sampling rate (200 kHz) significantly increased. However, the low pass filter usually attenuates events with a dwell time shorter than twice the filter rise time (2*Tr = 2*0.332/ f_c_ = 132 μs at f_c_ = 5 kHz or 6.6 μs at f_c_ = 100 kHz) [1]. Therefore, we conducted the ssDNA translocation experiments for bare substrates to investigate the effect of the filter.

Figure S11 shows the log-scaled histogram of the dwell times for 5.3 k-mer poly(dA) translocation using bare substrates. The data were measured using f_c_ = 100 kHz filter (upper) and f_c_ = 5 kHz filter (lower). In the 100-kHz filter data, the major part (82%) of the histogram was well-fitted to a single log-normal distribution and the characteristic dwell time (the peak position of the fitted curve) was 59 μs which is 9 times longer than 2*Tr. This result indicated that the measured events were not attenuated by the filter. The rest part exists as a long-tail in the histogram. It is considered that the interaction between ssDNA and sidewalls of the nanopore [2-5] contributes to these events with a long duration. When the cut-off frequency of the filter decreased from 100 kHz to 5 kHz, the dwell time of the most frequented events apparently increased from 59 μs to 250 μs. Although the events were significantly attenuated, the dwell time around 250 μs attributes to typical “non-interacted with beads” events when using 5-kHz filter.


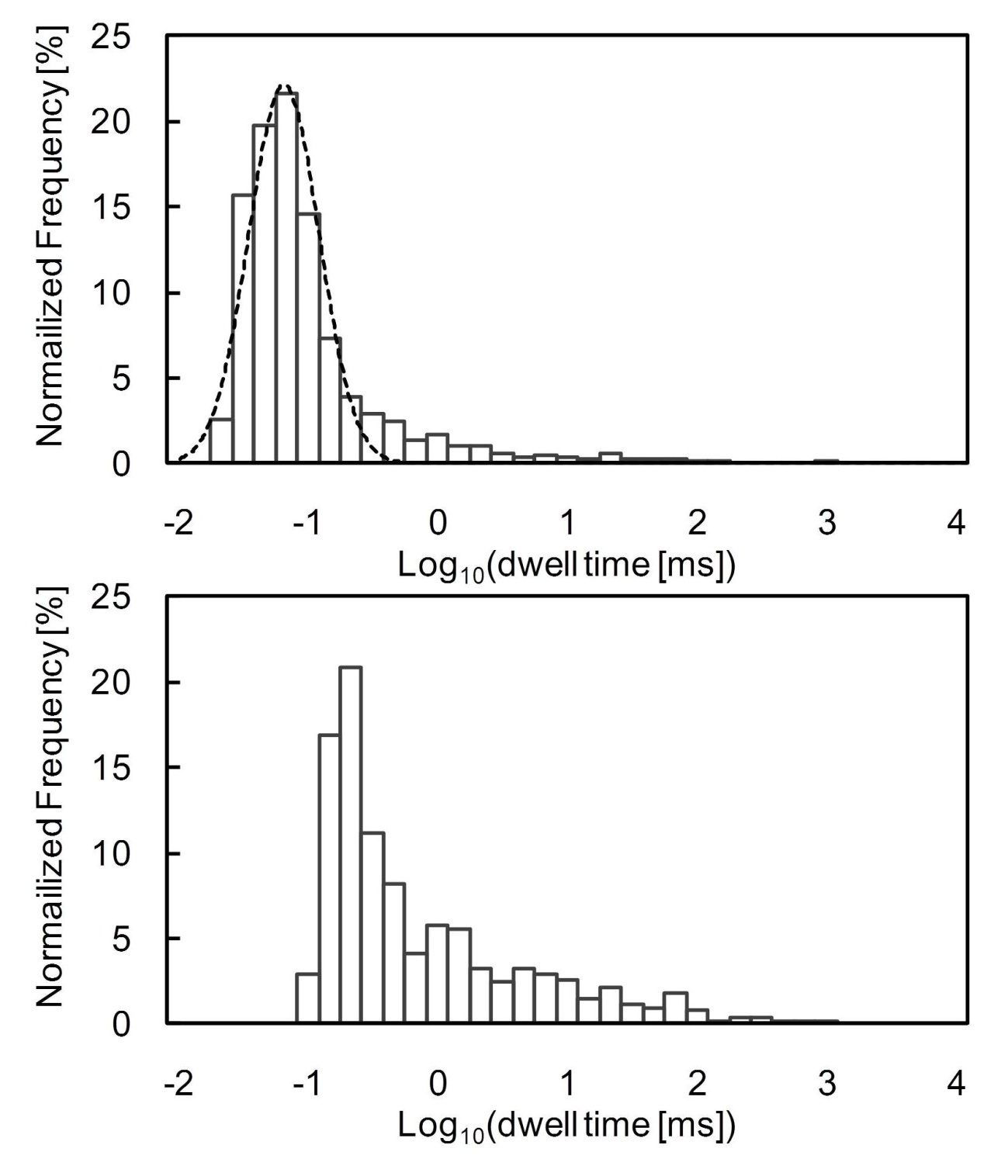


**Figure S11.** Log-scaled histogram of dwell time for 5.3 k-mer poly(dA) passing through a nanopore using bare substrates. The data was measured using 100 kHz filter (upper, N=2130) and 5 kHz filter (lower, N=608). Both measurement were performed under the same experimental condition (nanopore diameter: 2.0 nm, membrane thickness: 20 nm, voltage: 1 V). Broken line was a fitted curve using a log-normal distribution.

**SI-6. Data reproducibility**


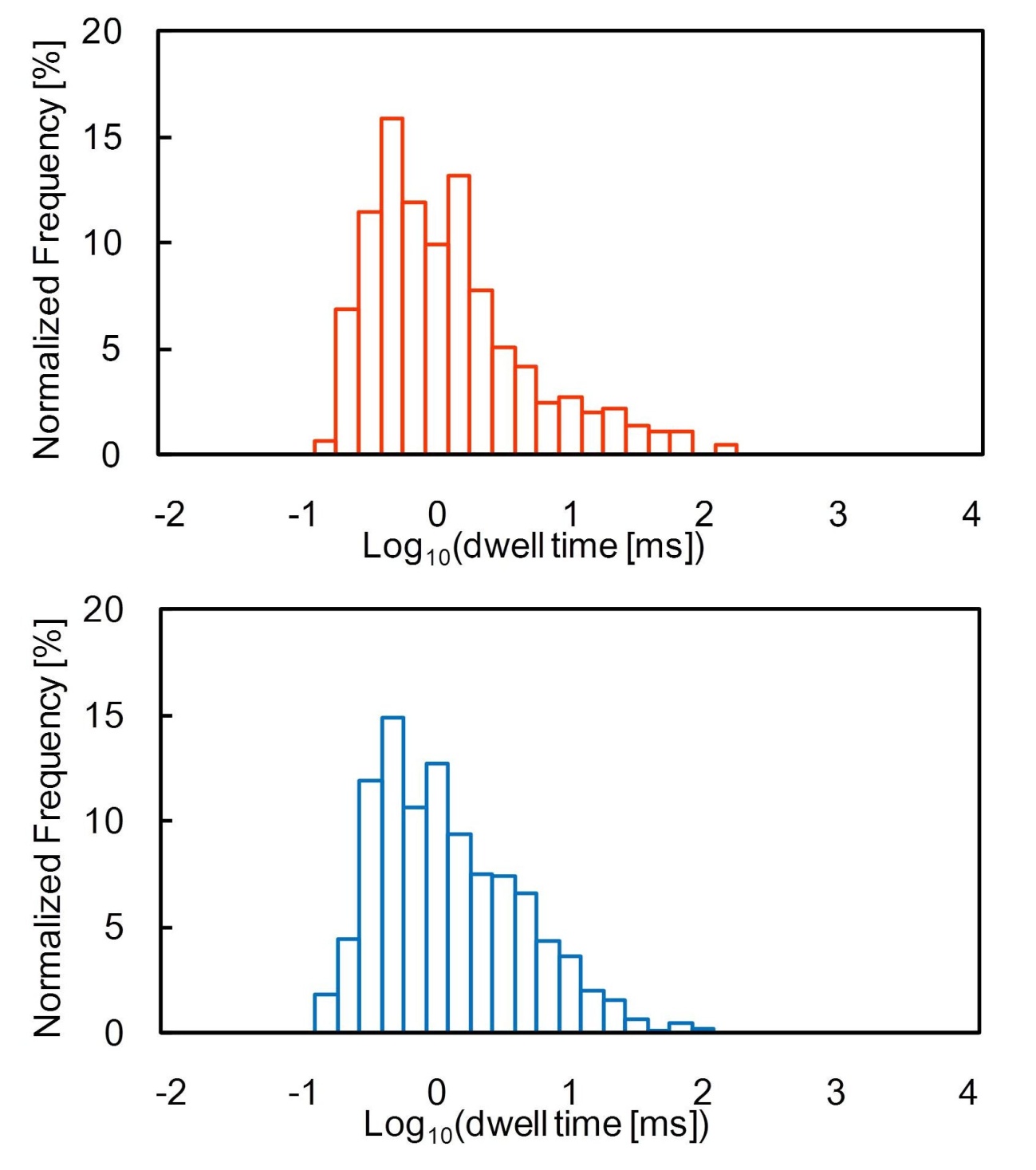


**Figure S12.** Data reproducibility of 60-mer poly(dA) translocation dwell time was confirmed using the 50-nm amine-silica bead-coated substrate. 1 st trial (upper, N=656) and 2 nd trial (lower, N=974) were performed at the same experimental condition (Nanopore diameter: 2.0 nm membrane thickness: 20 nm, Voltage: 1 V, low-pass filter: 5 kHz).

**References:**

[1] Plesa, C. *et al.* Fast translocation of proteins through solid state nanopores. *Nano Lett.* **13**, 658-663. (2013).

[2] Wanunu, M. *et al.* DNA translocation governed by interactions with solid-state nanopores. *Biophys. J.* **95**, 4716-4725 (2008).

[3] Van den Hout, M. *et al.* Distinguishable Populations Report on the Interactions of Single DNA Molecules with Solid-State Nanopores. *Biophys. J.* **99**, 3840−3848 (2010).

[4] Akahori, R. *et al.* Slowing single-stranded DNA translocation through a solid-state nanopore by decreasing the nanopore diameter. *Nanotechnology* **25**, 275501 (2014).

[5] Carson, S. *et al.* Smooth DNA transport through a narrowed pore geometry. *Biophys. J.* **107**, 2381-2393 (2014).
